# Stabilization and Synchronization of a Complex Hidden Attractor Chaotic System by Backstepping Technique

**DOI:** 10.3390/e23070921

**Published:** 2021-07-20

**Authors:** Jesus M. Munoz-Pacheco, Christos Volos, Fernando E. Serrano, Sajad Jafari, Jacques Kengne, Karthikeyan Rajagopal

**Affiliations:** 1 Faculty of Electronics Sciences, Benemérita Universidad Autónoma de Puebla, Puebla 72570, Mexico; 2 Laboratory of Nonlinear Systems, Circuits & Complexity (LaNSCom), Department of Physics, Aristotle University of Thessaloniki, 54124 Thessaloniki, Greece; volos@physics.auth.gr; 3 Instituto de Investigacion en Energia IIE, Universidad Nacional Autonoma de Honduras (UNAH), Tegucigalpa 11101, Honduras; feserrano@unitec.edu or; 4 Nonlinear Systems and Applications, Faculty of Electrical and Electronics Engineering, Ton Duc Thang University, Ho Chi Minh City 700000, Vietnam; sajad.jafari@tdtu.edu.vn; 5 Department of Electrical Engineering, University of Dschang, Dschang P.O. Box 134, Cameroon; kengnemozart@yahoo.fr; 6 Center for Nonlinear Systems, Chennai Institute of Technology, Chennai 600069, India; rkarthiekeyan@gmail.com or

**Keywords:** chaotic systems, hidden attractors, backstepping controller, synchronization, stabilization

## Abstract

In this paper, the stabilization and synchronization of a complex hidden chaotic attractor is shown. This article begins with the dynamic analysis of a complex Lorenz chaotic system considering the vector field properties of the analyzed system in the $C^{n}$ domain. Then, considering first the original domain of attraction of the complex Lorenz chaotic system in the equilibrium point, by using the required set topology of this domain of attraction, one hidden chaotic attractor is found by finding the intersection of two sets in which two of the parameters, *r* and *b*, can be varied in order to find hidden chaotic attractors. Then, a backstepping controller is derived by selecting extra state variables and establishing the required Lyapunov functionals in a recursive methodology. For the control synchronization law, a similar procedure is implemented, but this time, taking into consideration the error variable which comprise the difference of the response system and drive system, to synchronize the response system with the original drive system which is the original complex Lorenz system.

## 1. Introduction

Chaos theory, control and stabilization has been studied nowadays considering the vast amount of natural phenomena and physical systems in which chaos is present. Among these kinds of natural and physical systems, chaos is found in meteorological, astrophysics, and quantum systems, but beside these systems, chaos is found in engineering related systems such as electrical, mechanical, biological, and chemical systems. Complex variable chaotic systems represent phenomena found in optics and quantum systems, being a relevant topic that is worthy of investigation by scientists and researchers. Complex variable chaotic systems are mathematically more difficult to analyze than the real counterpart, remarking that chaotic systems are complex due to their nature and not necessarily if they are in the real and complex vector space, taking into consideration that the domain of attraction lies in the complex vector space $C^{n}$, considering that the limit cycles are found in this complex domain. One of the main discoveries of the representation of optic systems by complex variable chaotic systems is found in [1], in which the fundamental analogy of single-mode laser equation with the complex Lorenz chaotic system is derived. Basically, as explained in [2], a subcritical Hopfs bifurcation occurs in the Lorenz complex variable chaotic system in relation to real fluid dynamical processes. Besides, in papers like that in [3], it is shown that the Maxwell–Block equation is equivalent to the Lorenz system. The Maxwell–Block equation is obtained from a microscopic point of view in which this equation describes the interaction between an electromagnetic field and a two-atom assembly. From an optics framework, this electromagnetic field is treated as classical, including the reversibility with a 1/1 resonance. Experimentally, the Lorenz system is equivalent to the Maxwell–Block equation in the laser dynamics. Another important issue to consider in complex variable chaotic system is conservativeness, taking into consideration the energy conservation or dissipation in this kind of system due to the state variable flow is related to the stability of the system. There are important papers related to this research study that its worthy to mention considering that the divergence of the vector field of a chaotic system is used to find the system state variable. It is explained in this paper that by using the Bendixson theorem the hidden attractor is found. As explained in [4,5,6], conservative chaos is when the conservation of volume is constant in time, meanwhile, in the dissipative chaos case, the volume approaches zero as time goes to infinity. Therefore, in this paper, the hidden chaotic attractors are mostly considered dissipative, as they depend on the change in sign of the divergence of the dynamic system vector field, by finding the constant parameters in which the chaotic attractor is found. Another interesting result is found in [7], in which the fundamental laws of physics, as occurs in the dynamics of optical, mechanical, fluid, and celestial mechanics systems, the symmetry disappears due to dissipative phenomena which vary from a weak perturbation to strong dominant effects. These generic co-dominants instabilities are related with a resonance which occurs at zero or finite time frequencies. As it is known, hidden attractors are those kinds of chaotic systems in which the domain of attraction is outside the equilibrium points, meanwhile self-excited attractors are the kind of chaotic system in which the domain of attraction is related to the equilibrium points. Finding hidden attractors in complex variable chaotic systems is even more difficult than finding their real variable counterparts. However, similar to the real variable counterpart, by searching systematically, a domain of attraction outside the equilibrium points is crucial in order that hidden attractors which are not evident are found. Besides, the stabilization is not so easy, considering that it is necessary to implement a complex variable Lyapunov functional, or, in other words, to divide the real and integer part of the the chaotic system variables, taking into consideration that the stability conditions of complex variable systems are very similar to the real variable counterpart. For the synchronization case, it is essentially the same problem that in the stabilization case, but this problem is overcome by designing the appropriate Lyapunov functionals.

An example of complex variable chaotic systems can be found in [8], in which the stability of a complex variable uncertain chaotic system is analyzed. Then, in [9] a dual phase and a dual-antiphase synchronization of complex and real variable fractional order uncertain chaotic system is presented. Another example can be found in [10], where the synchronization of a time delayed complex variable system with discontinuous coupling is presented. Then, in [11] the multiple synchronization of three fractional order complex variable chaotic system is presented. Another interesting example can be found in papers like [12] in which the exponential synchronization of fractional order chaotic systems is presented. In this paper, a fractional order Lorenz and Lu systems are presented.

Hidden attractors has been widely studied nowadays, studies like that in [13] provide results in which the Lyapunov stability of a domain of a attraction of a new coupled chaotic system is presented. Then, in [14] nested, axisymmetric and centrosymmetric attractors are designed to create hidden multi-wing and multi-scroll chaotic attractors. Other example can be found in [15] in which hidden attractors and multistability are found and analyzed for a modified Chua circuit. In [16], the DSP implementation of a fractional order hidden and self excited attractor is evinced. Other novel results are found in papers like [17] in which hidden and self excited attractors are found in a Cournot oligopoly model.

The stabilization and synchronization of hidden attractors and self-excited chaotic systems is crucial for the purpose of this study. For example, in papers like [18] is shown a multi-stable four dimensional chaotic system with hidden and self-excited attractor in which an adaptive fuzzy sliding mode controller is implemented for synchronization purposes. Then, in [19] the backstepping control circuit design of a plasma torch chaotic system is presented. In [20] a new thermally excited chaotic jerk system with its adaptive backstepping control and circuit simulation is shown. Another example can be found in papers like [21] in which nonlinear resonant velocity feedback controller is used to stabilize self-excited vibrations in a nonlinear beam.

Before finalizing this section, we show some interesting results related to backstepping control, taking into consideration that this technique is so important in the control of mechanical, aeronautical, electrical, and, of course, chaotic systems. Some examples of backstepping control are found in papers [22] in which a backstepping controller for an air breathing hypersonic vehicle with mismatched uncertainties is presented. In [23], an output feedback backstepping control of hydraulic actuator is shown. Then, in [24] a feedback observer and backstepping control of chemical process is shown. Then an interesting example but with the application of backstepping control but in chaotic systems is found in [25], in which a backstepping sliding mode control for a Rossler chaotic system is presented. Finally, in [26] a fuzzy neural network backstepping control for a robotic system is presented.

In this paper, the stabilization and synchronization of complex hidden attractors by chaotic systems by a backstepping technique are proposed. The first part of this study deals in finding the hidden attractors in a complex Lorenz chaotic system. The hidden attractor is found by obtaining the required set topology in which the hidden attractor is found by implementing the Bendixson theorem. Therefore, other domains of attractions that are outside the equilibrium points are found with this methodology. As a novel contribution to the field, it is important to mention that the hidden attractor is found in the real and imaginary domains, something that has not been discovered yet. The phase portraits are presented to illustrate the behaviour of the complex chaotic system, evincing the periodic orbit of the studied complex Lorenz system. The backstepping stabilization and synchronization of this chaotic system is achieved by selecting extra variables and virtual inputs, that in the first case, the control law obtained by recursion stabilize the system in the equilibrium point, meanwhile in the second case, the error between the drive and response system approaches zero in finite time. One of the main contributions of this study regarding the design of a backstepping controller for stabilization and synchronization purposes, is that a complex variable backstepping controller is very odd to be reported in the literature, despite if this controller is for some sort of chaotic system or another complex dynamical system. For this reason considering the design of a complex variable Lyapunov function and virtual control inputs, the controller and synchronizer for the complex variable chaotic system is achieved. Finally, two numerical examples are provided with the respective discussion and conclusions.

## 2. Related Work

In this section, we give a short literature review related to hidden attractors and the control and synchronization of chaotic systems that is useful for this research study, and it is important not to omit them due to the direct relation that exists with the present work.

One of the important research studies found in the literature related to complex chaotic systems is found in [27], in which the projective synchronization of complex chaotic systems with known and unknown complex parameters is shown. The synchronization strategy shown in this study consists in the design of complex Lyapunov functions, something that has not been reported in the literature. Then, in [28] the synchronization of two time-delayed complex Lorenz chaotic systems is presented, research study that is important to mention because it provides the fundamental for the chaotic dynamic analysis of complex Lorenz chaotic systems. Then, important results are provided in [29], in which the hidden attractor and homoclinic orbits in a Lorenz like system which describe the fluid motion in a rotating cavity is presented. Then, in [30] a novel 3-D memristive time delay chaotic system with multi-scroll and hidden attractor is presented.

An example of backstepping control for chaotic systems can be found in [31], in which the control and synchronization of two coupled neurons is achieved. Then in [32] an optimal backstepping control for chaotic nonlinear systems is presented. Then in [33] the chaos control of a 4-D chaotic system using recursive backstepping control is evinced. Finally in [34] the synchronization of an antimonotonic hyper-jerk system and circuit realization is shown.

As mentioned in the previous section, there are other research studies that are important for this present work found in papers like [35] in which the backstepping synchronization and control of time delayed system is presented, meanwhile in [36] a multi-switching synchronization of nonlinear hyper-chaotic systems by backstepping control is presented. To finalize this section, in [37] the synchronization of a fractional order chaotic system is evinced. Other important references that must be cited in this research study paper; due to the importance of several control techniques for nonlinear, complex or chaotic systems are found in references like [38] in which a finite time tracking control for an uncertain multi input multi output MIMO system with input backlash is presented. In this paper modified finite time command filters are implemented in the design of a backstepping controller, then an error compensating mechanism is designed to reduce the filtering errors. Then in this paper a neuro-adaptive controller is implemented for the MIMO system with input backlash. Other interesting results are found in [39] another control technique when nonlinearities are found in the system are presented. In this paper a control strategy based on finite time is proposed for the stabilization of a switched system with hysteresis input. Levant differentiators are implemented to approximate the derivatives in the virtual control inputs. Then in this paper is shown that a finite time adaptive neural controller is implemented with a new command filter backstepping technique. Other control techniques for synchronization purposes not necessarily related to backstepping control are found in papers like [40] in which the dynamic of an atomic ensemble in a resonant cavity is analyzed with quasiperiodic route to chaos and its chaotic synchronization is shown. Meanwhile in [41] the synchronization of systems with nonlinear inertial coupling is achieved and evinced. Then in [42] the chaos control and anti-synchronization of a novel fractional order chaotic system is shown in which an adaptive sliding mode controller is implemented and shown in this article. Another interesting example can be found in [43] in which the chaos synchronization of stochastic time delayed reaction-diffusion neural networks are shown via output feedback control. Other interesting examples of chaos synchronization techniques are found in papers like [44] in which an interesting application is shown in which optical chaotic communication is achieved by correlation demodulation between two synchronized chaos lasers. Then in [45] the effects of control parameters on chaos synchronization by optical feedback is presented. Finally, other interesting papers are shown in [46,47], where the synchronization of an uncertain fractional order chaotic system is presented and the chaos synchronization of nonlinear pharmacological system is presented, respectively.

## 3. Problem Formulation: Complex Hidden Attractor

In this section, the establishment of the complex variable Lorenz chaotic system is presented, along with a systematical methodology to find the hidden attractor in this analyzed system is provided. First, note that the complex variable Lorenz chaotic system is provided for a dynamical analysis in order to obtain the region of attraction taking into consideration when this region is ubicated in the equilibrium point of the system. In opposition to real chaotic systems, when complex variables are found in the analyzed system, it is more difficult to find hidden attractors. Therefore, for this purpose, the Bendixson theorem is needed, a theorem that is related to the energy dissipation of the system, by finding separately the set topology for the real and imaginary part of the proposed Lorenz system. The Bendixson theorem, as shown as follows, is important to find the region in which the hidden attractor is found.

**Theorem** **1.** *Ref. [48] Consider the following nonlinear system:*

(1) $$\begin{array}{ccc} {\dot{x}}_{1} & = & f_{1}\left(x_{1}\left(t\right),x_{2}\left(t\right)\right)\\ {\dot{x}}_{2} & = & f_{2}\left(x_{1}\left(t\right),x_{2}\left(t\right)\right) \end{array}$$

*With $x_{1}\left(0\right)=x_{10}$ and $x_{2}\left(0\right)=x_{20}$. If there exist a vector field $f:D\to R^{2}$ in which there are not equilibrium points in D. If ∇.f(x)≠0 and ∇.f(x) does not change sign in D, then system (1) has no periodic orbits in D.*

Therefore, to find the hidden attractor in a Lorenz chaotic system, consider the following complex Lorenz system [27]:

(2) $$\begin{array}{ccc} \dot{x} & = & \sigma (y-x)\\ \dot{y} & = & rx-xz-y\\ \dot{z} & = & \frac{1}{2}\left(\bar{x}y+x\bar{y}\right)-bz \end{array}$$

In which x,y∈C and z∈R such as $x=x_{r}+x_{i}j$, $y=y_{r}+y_{i}j$ and $z=z_{r}$. The complex conjugate of *x* and *y* is denoted by $\bar{x}$ and $\bar{y}$, respectively, and as it is known, $j=\sqrt{-1}$. Now, system (2) is divided into a real and imaginary part as shown next:

(3) $$\begin{array}{ccc} {\dot{x}}_{r} & = & \sigma (y_{r}-x_{r})\\ {\dot{y}}_{r} & = & rx_{r}-x_{r}z_{r}-y_{r}\\ {\dot{z}}_{r} & = & x_{r}y_{r}+x_{i}y_{i}-bz_{r} \end{array}$$

(4) $$\begin{array}{ccc} {\dot{x}}_{i} & = & \sigma (y_{i}-x_{i})\\ {\dot{y}}_{i} & = & rx_{i}-x_{i}z_{r}-y_{i} \end{array}$$

In order to find the hidden attractors from (3) and (4), and by using Theorem 1, consider the following sets:

(5) $$\begin{array}{ccc} A_{R}\left(X\right) & = & \left\{X\in C^{n}:\nabla .Real\left(f\left(X\right)\right)\leq 0\right\}\\ A_{I}\left(X\right) & = & \left\{X\in C^{n}:\nabla .Im\left(f\left(X\right)\right)\leq 0\right\} \end{array}$$

In which $X={\left[x,y,z\right]}^{T}$ and with the following vector field:

(6) $$f\left(x\right)=\left[\begin{array}{c} \sigma (y-x)\\ rx-xz-y\\ \frac{1}{2}\left(\bar{x}y+x\bar{y}\right)-bz \end{array}\right]=\left[\begin{array}{c} f_{1}\left(X\right)\\ f_{2}\left(X\right)\\ f_{3}\left(X\right) \end{array}\right]$$

Basically, with the sets (5), the following set topology operation is necessary to obtain the region of attraction of the hidden chaotic attractor of the complex variable Lorenz system [49]:

(7) $$A\left(X\right)=A_{R}\left(X\right)⋂A_{I}\left(X\right)$$

It is important in this case that the parameter *r* is kept constant, and while varying the parameters σ and *b*, the region of attraction is obtained. Therefore, to find a hidden chaotic attractor on the Lorenz system from (7), the following regions for the parameters σ and *b* are found: σ≥−1 and b≥−σ−1, respectively.

**Figure 1:**
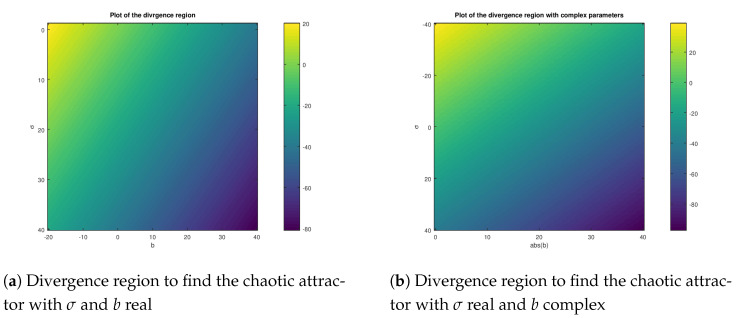
Divergence region of the complex variable Lorenz system in which the hidden attractor is found while varying *σ* and *b*.

In Figure 1a, the divergence region of the set A(X) is shown for real parameter values. Note that the regions in which the hidden attractor for the complex Lorenz chaotic system can be found in the dark blue area while keeping constant the parameter *r* constant with r=35. Remember from the Bendixson theorem (Theorem 1) that the system does not have periodic solution in D when the gradient of the vector field f(X) does not change sign in such set, so basically, the hidden attractors are found in the transition from the positive value of the gradient to the negative value as shown in Figure 1a, in other words, in the transitions from the green area to the dark blue area. It is important to remark that at least one hidden attractor was found on that region, but other hidden attractors can be found on that area. Meanwhile, in Figure 1b, one complex parameter is selected to find the transition in the divergence plot in which hidden chaotic attractors are found. The selected complex value parameter selected in this case is the parameter *b*, and while varying this parameter the hidden chaotic attractor region is found in the transition from the positive to negative value of the divergence. As it is noticed, the hidden attractor is not only found for real valued parameter, it can be found also for complex valued parameters considering the absolute value of the complex parameter. This occurs because the region of attraction is in the complex $C^{n}$ plane, independently if the parameters are real or complex, something important in the design of the stabilization and synchronization controller.

**Figure 2:**
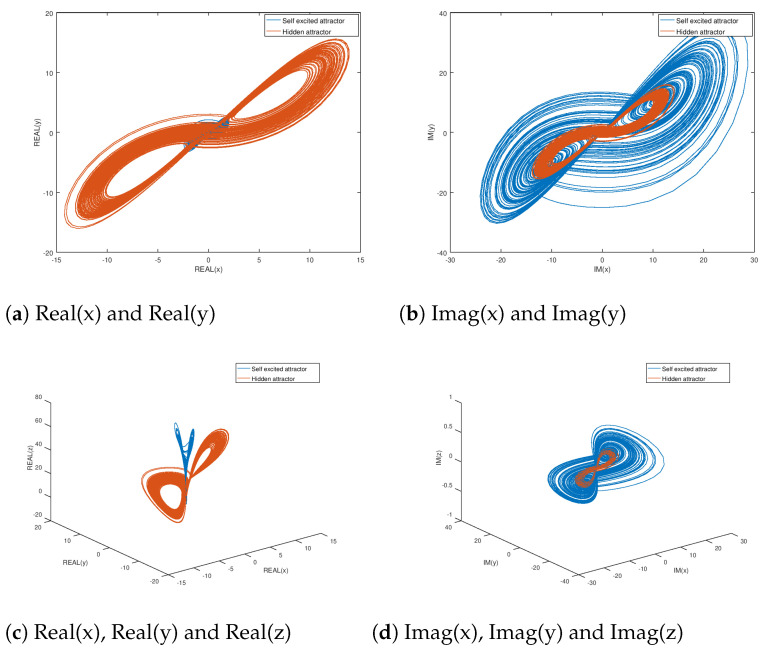
Phase portrait plots of the original and hidden attractors for the complex variable Lorenz chaotic system.

**Figure 3:**
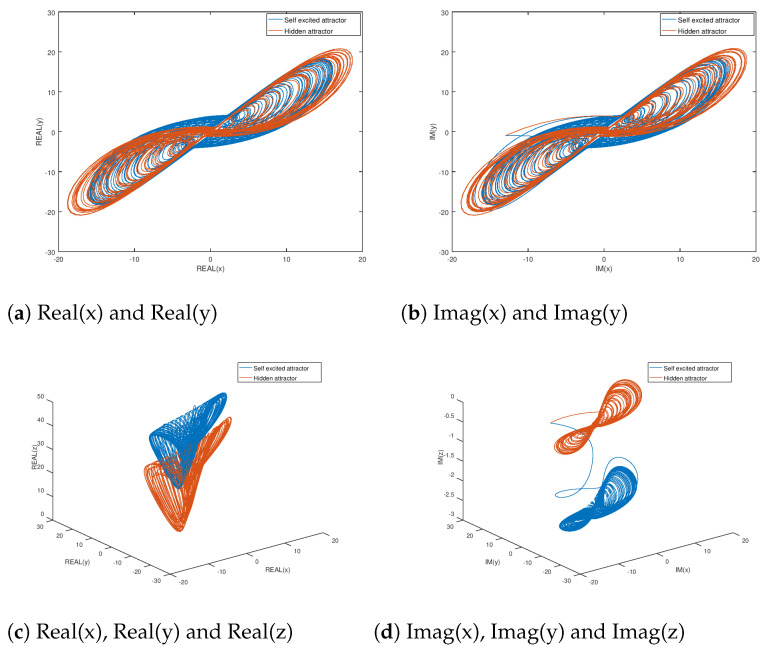
Phase portrait plots of the original and hidden attractors for the complex variable Lorenz chaotic system with complex parameter *b* = 2.7 + 0.2*j* for the self-excited attractor and *b* = 4.9365 + 0.1*j* for the hidden attractor.

Meanwhile, in Figure 2 the phase portraits of the original and hidden attractor found by the previous mentioned methodology are shown. The parameters in which the hidden attractor are found are σ=20, r=21, and b=4. The divergence region in which σ and *b* are found can be identified as shown in Figure 1, and they are used later in the numerical experiment section for the stabilization and synchronization of the complex variable Lorenz hidden chaotic attractor. It can be noticed in Figure 2 that the obtained hidden chaotic attractor reaches a different domain of attraction in comparison with the original domain of attraction, and as explained before, other hidden attractors can be found, but in this paper at least one hidden attractor is found. Meanwhile, in Figure 3, the phase portraits of the self excited and hidden chaotic attractor are shown with the system parameters σ=14, r=35, and b=2.7+0.2j, for the self-excited attractor, and σ=20, r=21, and b=4.9365+0.1j for the hidden chaotic attractor. The behaviour of the chaotic attractors could be verified in these figures when there is a complex valued parameter, observing how the hidden attractors are found in regions of attraction that are different from the equilibrium points. This objective is achieved by selecting the appropriate parameters from the divergence region as explained before. Something important to remark is that all the chaotic system parameters could be complex valued, but for parameter selection purposes, in this study only one parameter is complex.

## 4. Control and Synchronization of the Hidden Attractor

In this section, two novel backstepping control strategies are presented for the stabilization and synchronization of the hidden attractors found in the complex variable Lorenz chaotic system. For the stabilization case, an extra variable is established, and then by establishing the required Lyapunov functionals, the control laws for the system are found by the implementation of a virtual control input and by a recursive procedure. Something similar occurs for the synchronization case. The synchronization case consists first in implementing as a drive system the original attractor of the Lorenz complex system, and the response system consists in the hidden attractor found in this research study. Then an extra variable is established, in which the error variable between the drive and response states, is took into consideration and selecting the required Lyapunov functionals to find the control synchronization laws by the use of virtual control inputs and by a recursive procedure.

### 4.1. Stabilization of the Complex Hidden Attractor

For stabilization purposes, consider the following controlled complex variable Lorenz system:

(8) $$\begin{array}{ccc} {\dot{x}}_{1} & = & f_{1}+u_{1}\\ {\dot{x}}_{2} & = & f_{2}+u_{2}\\ {\dot{x}}_{3} & = & f_{3}+u_{3} \end{array}$$

In which $f_{1}$, $f_{2}$, and $f_{3}$ are defined in (6), and $u_{1}$, $u_{2}$, and $u_{3}$ are the control inputs. Now the following extra states are considered for the backstepping recursive design:

(9) $$\begin{array}{ccc} z_{1} & = & x_{1}\\ z_{2} & = & x_{2}+\alpha_{1}\\ z_{3} & = & x_{3}+\alpha_{2} \end{array}$$

In which $\alpha_{1}$ and $\alpha_{2}$ are the virtual control inputs, defined later. In order to design the backstepping controller, consider the following Lyapunov function:

(10) $$V_{1}=\frac{1}{2}z_{1}^{2}$$

Now, taking the derivative of (10), yields

(11) $${\dot{V}}_{1}=z_{1}{\dot{z}}_{1}=z_{1}\left(f_{1}+u_{1}\right)$$

Then by making $u_{1}=-f_{1}-z_{1}$, (11) becomes ${\dot{V}}_{1}=-z_{1}^{2}$. Now, consider the following Lyapunov function:

(12) $$V_{2}=V_{1}+\frac{1}{2}z_{2}^{2}$$

Now, taking the derivative of (12), yields

(13) $$\begin{array}{ccc} {\dot{V}}_{2} & = & -z_{1}^{2}+z_{2}\left({\dot{x}}_{2}+{\dot{\alpha }}_{1}\right)\\ {\dot{V}}_{2} & = & -z_{1}^{2}+z_{2}\left(f_{2}+u_{2}+{\dot{\alpha }}_{1}\right) \end{array}$$

Then by making $u_{2}=-f_{2}-z_{2}$ and ${\dot{\alpha }}_{1}=z_{2}^{-1}z_{1}^{2}$ the Lyapunov function derivative (13) becomes in ${\dot{V}}_{2}=-z_{2}^{2}$. Defining the following Lyapunov functional:

(14) $$V_{3}=V_{1}+V_{2}+\frac{1}{2}z_{3}^{2}$$

Then, by taking the time derivative of the Lyapunov function (14) yields:

(15) $$\begin{array}{ccc} {\dot{V}}_{3} & = & -z_{1}^{2}-z_{2}^{2}+z_{3}\left({\dot{x}}_{3}+{\dot{\alpha }}_{2}\right)\\ {\dot{V}}_{3} & = & -z_{1}^{2}-z_{2}^{2}+z_{3}\left(f_{3}+u_{3}+{\dot{\alpha }}_{2}\right) \end{array}$$

Now by substituting the following control input $u_{3}=-f_{3}-z_{3}$ and ${\dot{\alpha }}_{2}=-z_{3}$ and substituting in (15) yields:

(16) $${\dot{V}}_{3}=-z_{1}^{2}-z_{2}^{2}-2z_{3}^{2}\leq 0$$

So with (16) the stability of the system is ensured.

### 4.2. Synchronization of the Complex Hidden Attractor

For the backstepping synchronization of the drive and response system, consider the following systems:

(17) $$\begin{array}{ccc} {\dot{x}}_{1d} & = & f_{1d}\\ {\dot{x}}_{2d} & = & f_{2d}\\ {\dot{x}}_{3d} & = & f_{3d} \end{array}$$

(18) $$\begin{array}{ccc} {\dot{x}}_{1r} & = & f_{1r}+u_{1}\\ {\dot{x}}_{2r} & = & f_{2r}+u_{2}\\ {\dot{x}}_{3r} & = & f_{3r}+u_{3} \end{array}$$

with the following error variables and its derivatives:

(19) $$\begin{array}{ccc} e_{i} & = & x_{ir}-x_{id}\\ {\dot{e}}_{i} & = & {\dot{x}}_{ir}-{\dot{x}}_{id}\\ {\dot{e}}_{i} & = & \underset{f_{ie}}{\underbrace{f_{ir}-f_{id}}}+u_{i} \end{array}$$

Then, consider the following extra variables for synchronization purposes:

(20) $$\begin{array}{ccc} z_{1} & = & e_{1}\\ z_{2} & = & e_{2}+\alpha_{1}\\ z_{3} & = & e_{3}+\alpha_{2} \end{array}$$

So in order to obtain the backstepping control laws consider the following Lyapunov functional:

(21) $$V_{1}=\frac{1}{2}z_{1}^{2}$$

Now obtaining the first derivative of (21) yields

(22) $${\dot{V}}_{1}=z_{1}{\dot{z}}_{1}=z_{1}\left(f_{1e}+u_{1}\right)$$

Now by selecting the control input $u_{1}=-f_{1e}-k_{1}z_{1}$ for $k_{1}\in R^{+}$ making (22) ${\dot{V}}_{1}=-k_{1}z_{1}^{2}$.

Now by selecting the following Lypunov functional:

(23) $$V_{2}=V_{1}+\frac{1}{2}z_{2}^{2}$$

Now by obtaining the first derivative of (23) is obtained:

(24) $${\dot{V}}_{2}=-k_{1}z_{1}^{2}+z_{2}\left(f_{2e}+u_{2}+{\dot{\alpha }}_{1}\right)$$

Then by making the control input $u_{2}=-f_{2e}-k_{2}z_{2}$ and ${\dot{\alpha }}_{1}=k_{1}z_{2}^{-1}z_{1}^{2}$ the first derivative of the Lyapunov function (24) becomes in ${\dot{V}}_{2}=-k_{2}z_{2}^{2}$ where $k_{2}\in R^{+}$. Now by selecting the third and last Lyapunov function:

(25) $$V_{3}=V_{1}+V_{2}+\frac{1}{2}z_{3}^{2}$$

Now, by taking the time derivative of (25) the following result is obtained:

(26) $${\dot{V}}_{3}=-k_{1}z_{1}^{2}-k_{2}z_{2}^{2}+z_{3}\left(f_{3e}+u_{3}+{\dot{\alpha }}_{2}\right)$$

Now by making the control input $u_{3}=-f_{3e}-z_{3}$ and ${\dot{\alpha }}_{2}=-k_{3}z_{3}$ with $k_{3}\in R^{+}$ the following result is obtained:

(27) $${\dot{V}}_{3}=-k_{1}z_{1}^{2}-k_{2}z_{2}^{2}-\left(1+k_{3}\right)z_{3}^{2}\leq 0$$

So the backstepping design procedure is finished by ensuring the system stability.

## 5. Numerical Experiments

In this section, three numerical experiments are done to validate the theoretical results obtained in this study. The first numerical experiment consists in the stabilization of the analyzed Lorenz chaotic system. Meanwhile, the second numerical experiment consists in the synchronization of two identical Lorenz chaotic system. Finally, the third experiment consists in the synchronization of a Lorenz hidden chaotic attractor with a complex valued parameter. The parameters for the drive and response system are σ=14, r=35, and b=3.7 for system (17); meanwhile, for system (18), the parameters are σ=20, r=21 and b=4. The initial conditions for the drive system (17) are $X_{d}\left(0\right)={\left[-0.1-0.01j,0.01-0.01j,0.01\right]}^{T}$. Furthermore, the initial conditions for the response system are $X_{r}\left(0\right)={\left[-5-5j,0.1-0.1j,0.1\right]}^{T}$. These parameters are mostly for the experiments 1 and 2. The parameters and initial conditions for the experiment 3, are σ=14, r=35 and b=2.7+0.2j with initial conditions $X_{d}\left(0\right)={\left[0.01+13j,0.01+j,35\right]}^{T}$ in the case of the drive system. Besides, the parameters of the response system are σ=20, r=35 and b=4.9365+0.1j with initial conditions $X_{r}\left(0\right)={\left[0.01+13j,0.01+j,35\right]}^{T}$.

### 5.1. Experiment 1: Stabilization of the Hidden Attractor

The objective in this experiment is to validate the backstepping controller for the stabilization of the complex variable Lorenz chaotic system (18) with inputs. As it is verified, the system variables in their real and imaginary components are driven to the equilibrium points from the domain of attraction of the hidden attractor. It can be verified that the backstepping controller performance overcome this stabilization problem, and the chaos suppression is done in a very efficient manner.

**Figure 4:**
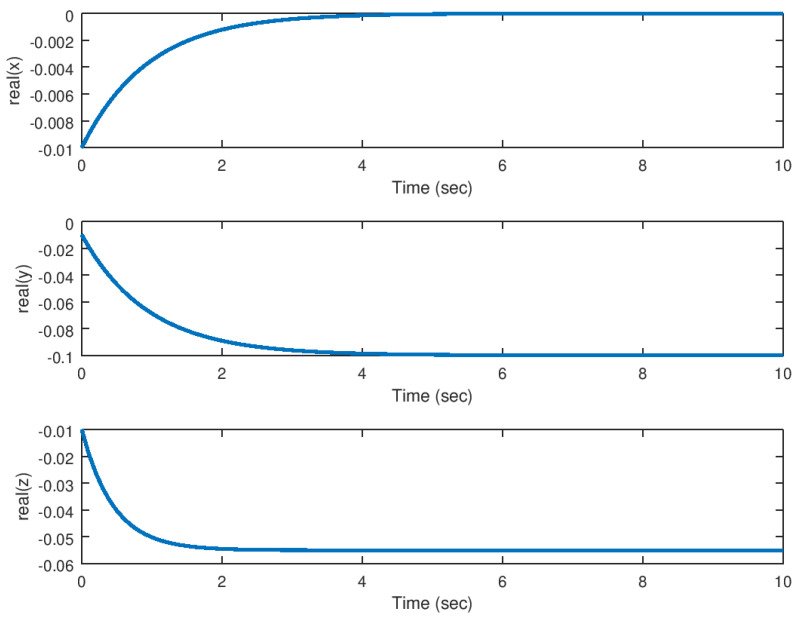
Evolution in time of the real part of the state variables in the stabilization problem.

**Figure 5:**
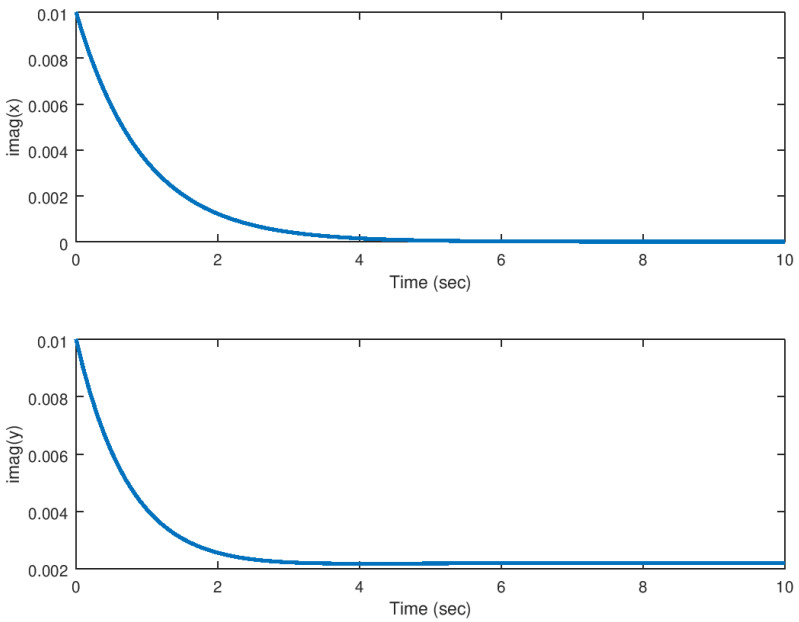
Evolution in time of the imaginary part of the state variables in the stabilization problem.

In Figure 4 and Figure 5 it can be noticed how these variables are driven to the equilibrium in finite time, while suppressing the chaotic behaviour in this system. As explained before, it is important to notice that these variables are driven from the domain of attraction of the hidden attractor to the equilibrium point of the original system in a very efficient manner. The time response of the backstepping controller is considerably good and there are not other effects like unwanted oscillations or peaks in the system variables response, something desirable for an application in physical systems.

**Figure 6:**
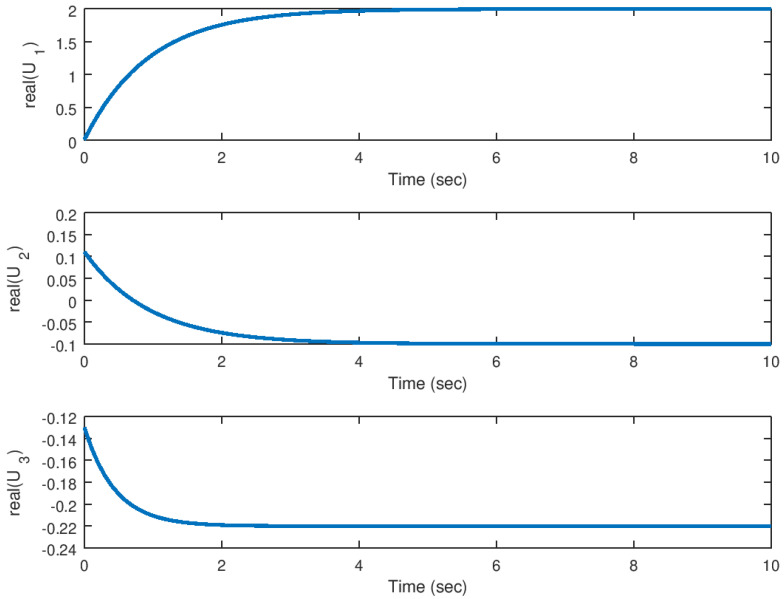
Evolution in time of the real part of the input variables in the stabilization problem.

**Figure 7:**
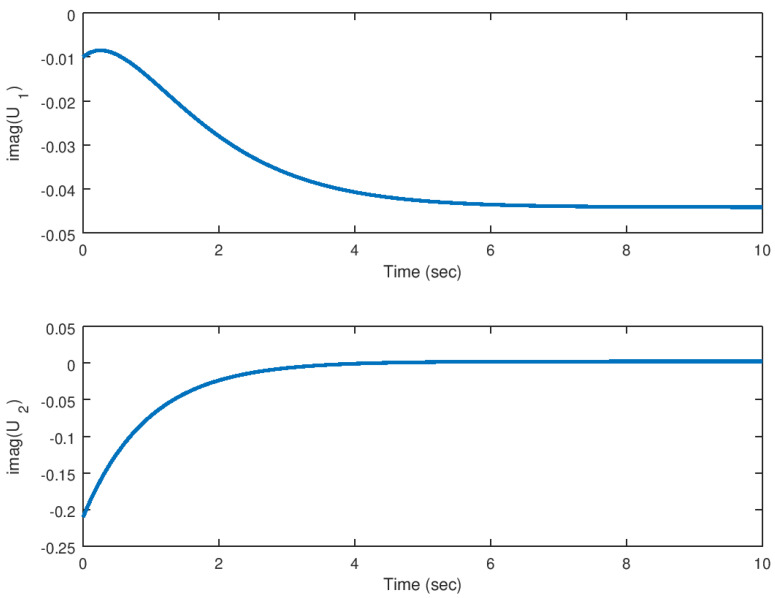
Evolution in time of the imaginary part of the input variables in the stabilization problem.

Meanwhile, in Figure 6 and Figure 7, the control input response in its real and imaginary components are shown. It is verified that the control effort generated by the backstepping controller inputs is considerably small taking into consideration that the theoretical analysis done in this study is only for mathematical purposes, but something that is desirable in a real application system. Unwanted oscillation are suppressed in the control inputs something important to avoid unwanted results in the system response.

### 5.2. Experiment 2: Synchronization of the Hidden Attractor with Real Parameter

In this subsection a numerical experiment is performed in which the drive system (17) has the original domain of attraction of the complex variable Lorenz chaotic system, which drive the response system (18), which is in the hidden attractor domain of attraction, in order that the error between the drive system variables states and the response system variables states to zero in finite time. Similar to the stabilization case, this experiment is intended to drive the hidden chaotic domain of attraction to the equilibrium points (the domain of attraction of the original complex variable Lorenz system) as corroborated in this numerical example, taking into consideration that despite that this is only a theoretical research but considers the physical implications of the results presented in this paper.

**Figure 8:**
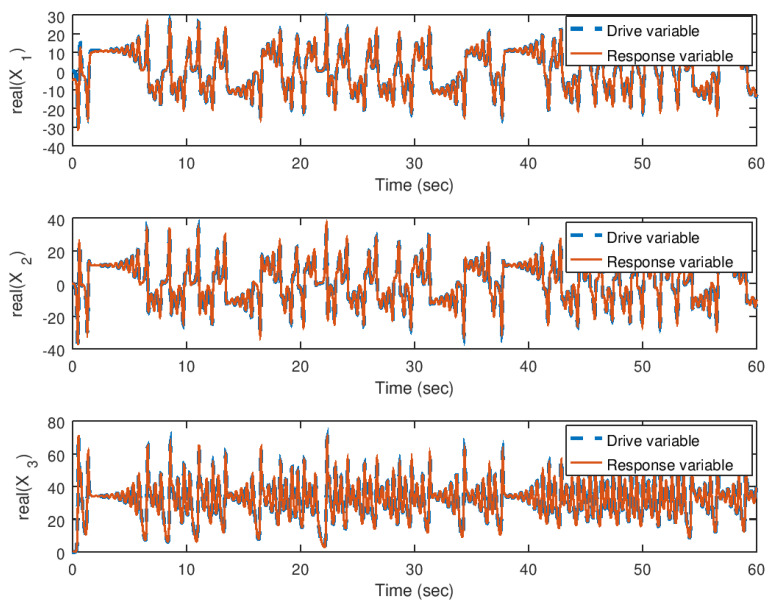
Evolution in time of the real part of the state variables in the synchronization problem.

**Figure 9:**
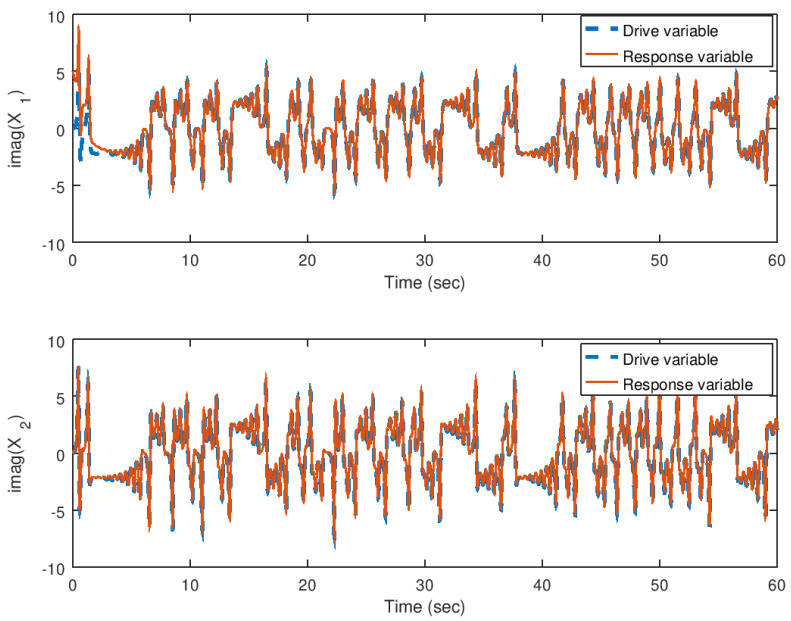
Evolution in time of the imaginary part of the state variables in the synchronization problem.

The results obtained in this numerical experiments depicts in Figure 8 and Figure 9 that the synchronization achieved by the backstepping controller is done in a faster and accurate way, as it is corroborated later, due to the controller action acts in a very efficient way for the real and imaginary parts of the response state variables. The synchronization between these systems, even when the systems has the same structure, the domains of attractions differs so the domain of attraction of the hidden attractor is driven to the desired equilibrium points of the drive system.

**Figure 10:**
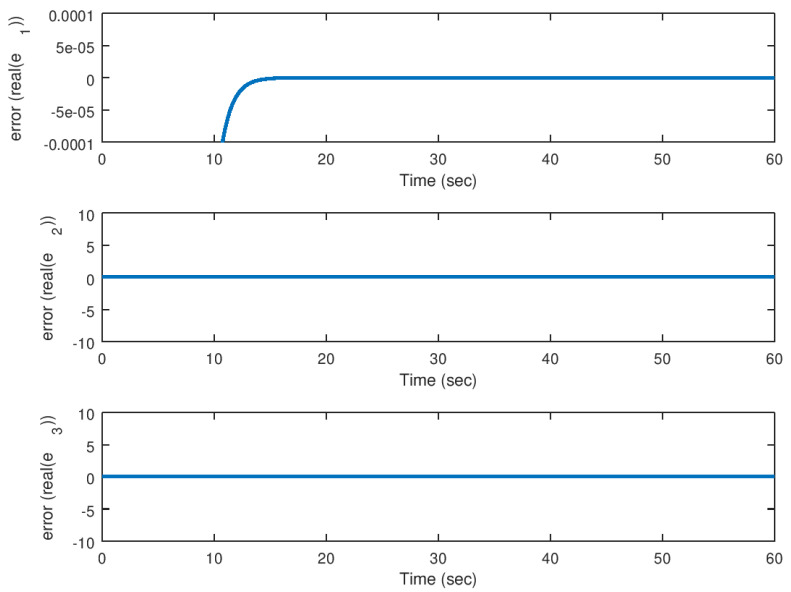
Evolution in time of the real part of the error variables in the synchronization problem.

**Figure 11:**
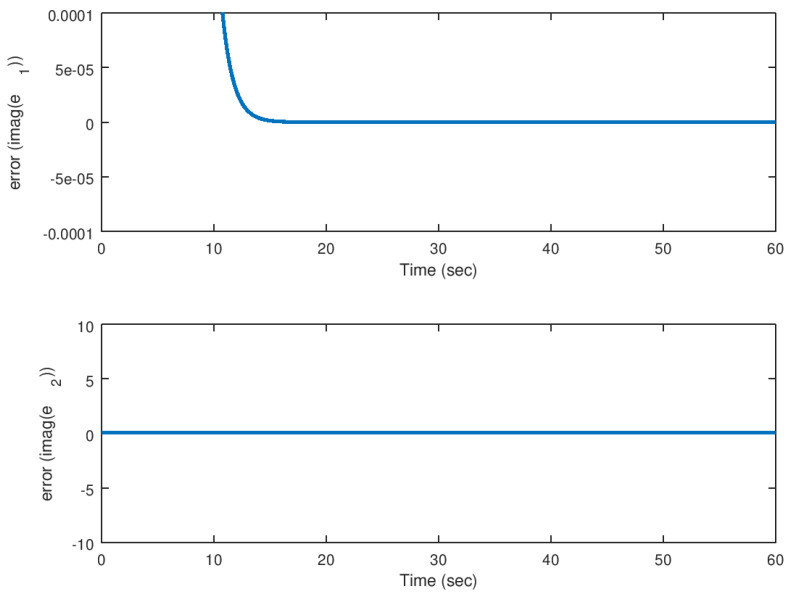
Evolution in time of the imaginary part of the error variables in the synchronization problem.

Then, in Figure 10 and Figure 11, the evolution in time of the error variables for the real and imaginary parts of the complex variable Lorenz chaotic system is shown. It is corroborated in these figures that the error between the synchronized state variables reach the origin in finite time.

**Figure 12:**
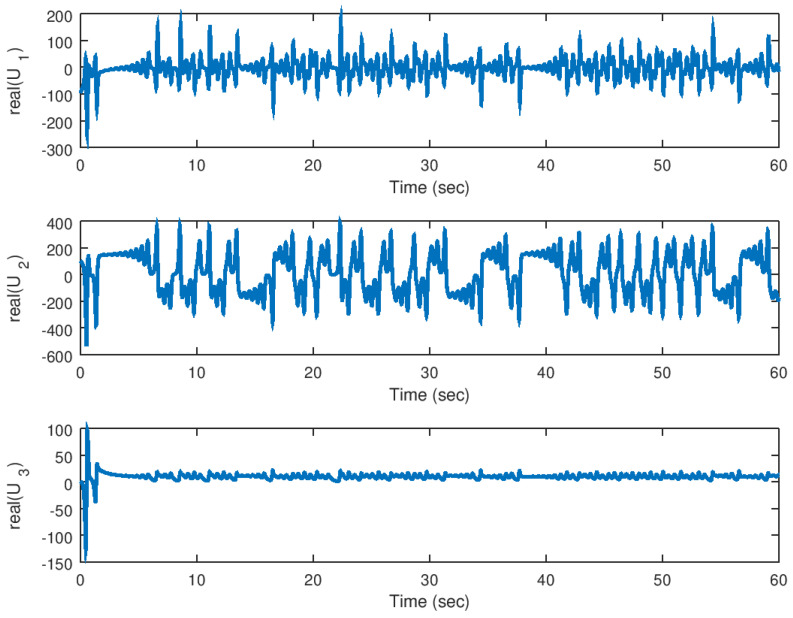
Evolution in time of the real part of the input variables in the synchronization problem.

**Figure 13:**
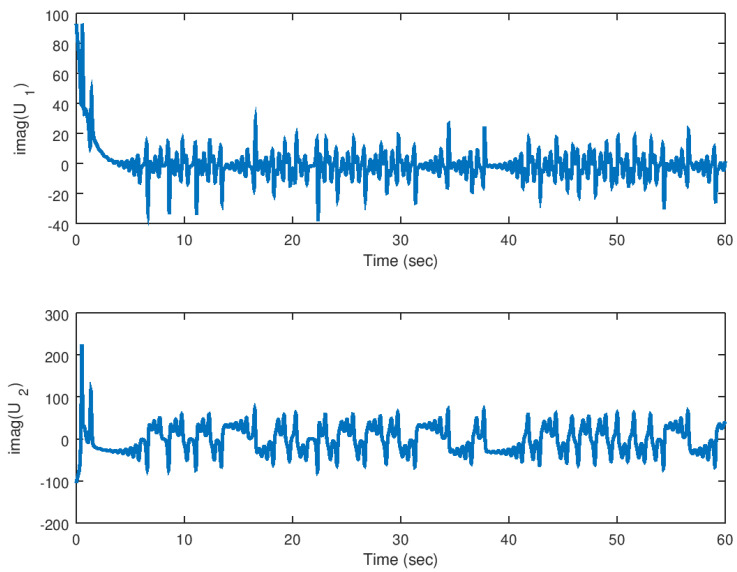
Evolution in time of the imaginary part of the input variables in the synchronization problem.

Finally, in Figure 12 and Figure 13, the real and imaginary parts of the input variables for the response system are shown, in which it is noticed that independently of the complexity of the synchronized system the desired control response is provided in order to make the desired error variables to reach the origin in finite time.

### 5.3. Experiment 3: Synchronization of the Hidden Attractor with Complex Value Parameter

In this experiment, the chaotic system which possesses the hidden attractor is considered as a response system, while the chaotic system with the self excited attractor is used as a drive system. As explained in the beginning of this section, both response and drive systems are experimented with the complex valued parameter *b*. The reason is to validate, as explained in theoretical results obtained in this study, that the proposed backstepping synchronization controller can be implemented when complex valued parameters are considered. In this experiment only one parameter is complex valued but two or the three parameters of this chaotic system can be complex valued.

**Figure 14:**
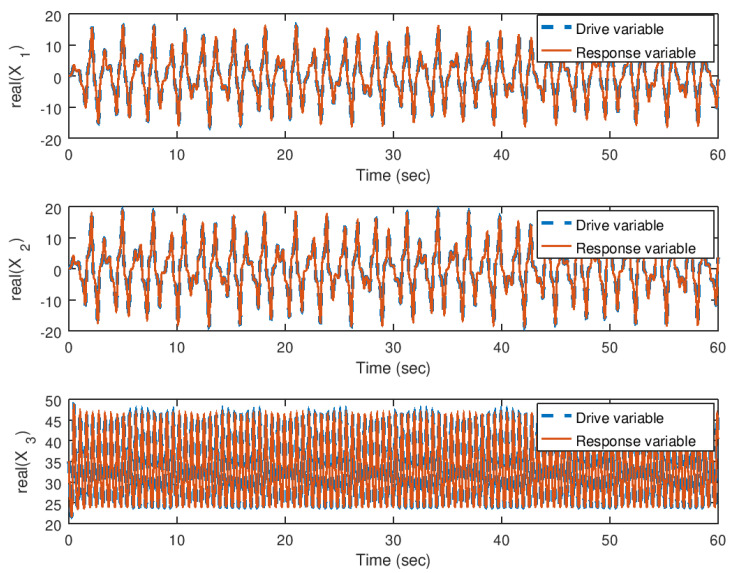
Evolution in time of the real part of the state variables in the synchronization problem with complex parameter.

**Figure 15:**
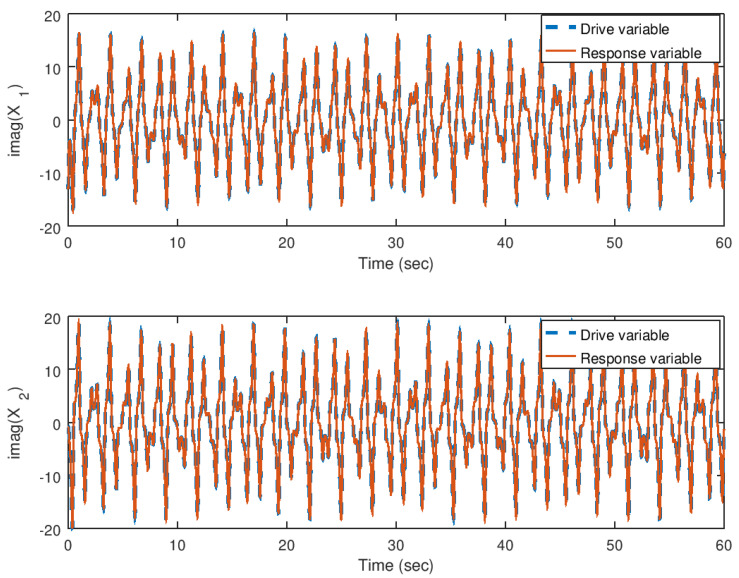
Evolution in time of the imaginary part of the state variables in the synchronization problem with complex parameter.

In Figure 14 and Figure 15, the evolution in time of the state variables of the drive and response system are shown in their real and imaginary parts, respectively. It can be noticed that the synchronization is achieved faster by the implementation of the backstepping synchronization controller, independently if the parameter *b* is complex. As explained before, due to the design of the backstepping controller with a complex variable Lyapunov function, allows to implement one or all the parameters with complex values.

**Figure 16:**
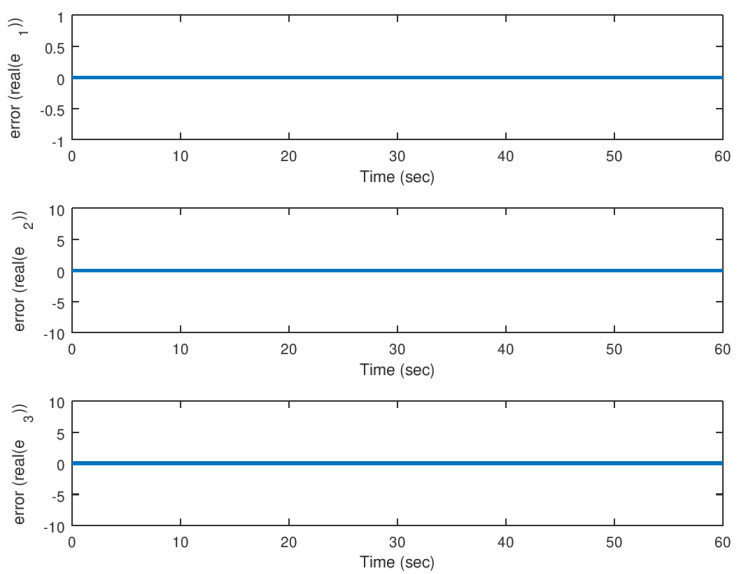
Evolution in time of the real part of the error variables in the synchronization problem with complex parameter.

**Figure 17:**
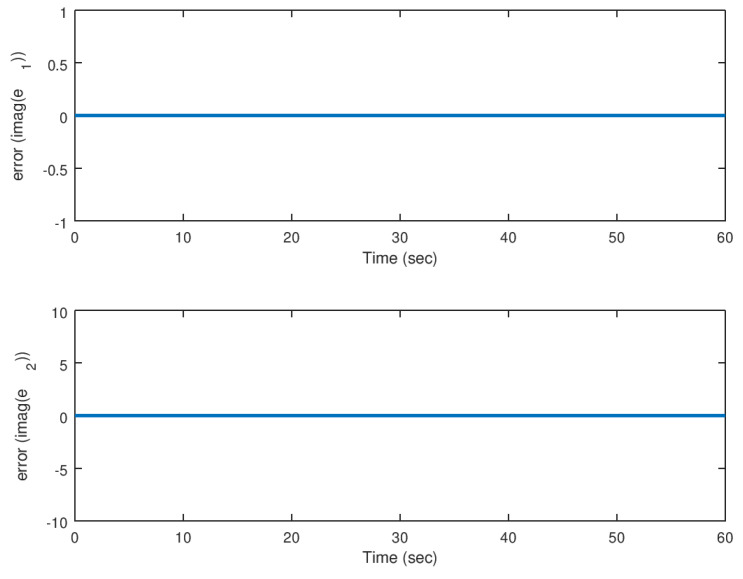
Evolution in time of the imaginary part of the error variables in the synchronization problem with complex parameter.

On the other hand, in Figure 16 and Figure 17, the error variables, in other words, the difference between the drive and response variables, for their real and imaginary parts, respectively, are shown. As corroborated before, the error reaches the origin faster due to the action of the backstepping controller independently of the complex valued parameter *b*. It is corroborated also, that similar to the real valued parameters, the synchronization is achieved satisfactorily.

**Figure 18:**
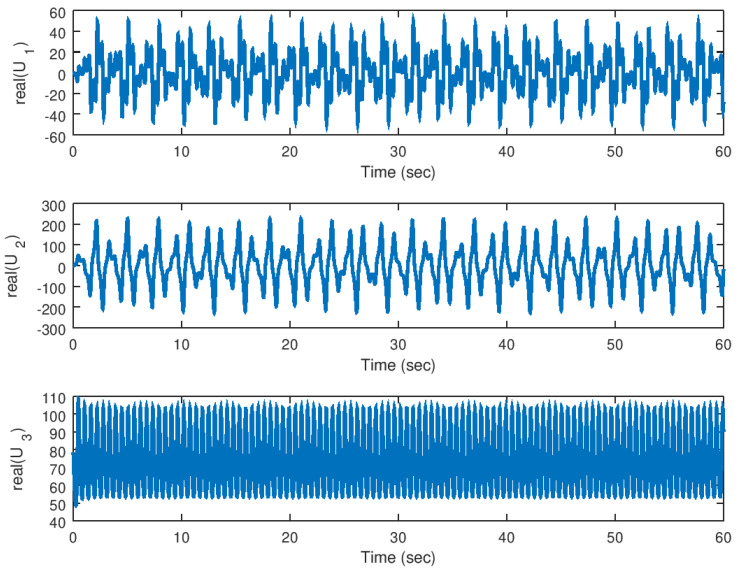
Evolution in time of the real part of the input variables in the synchronization problem.

**Figure 19:**
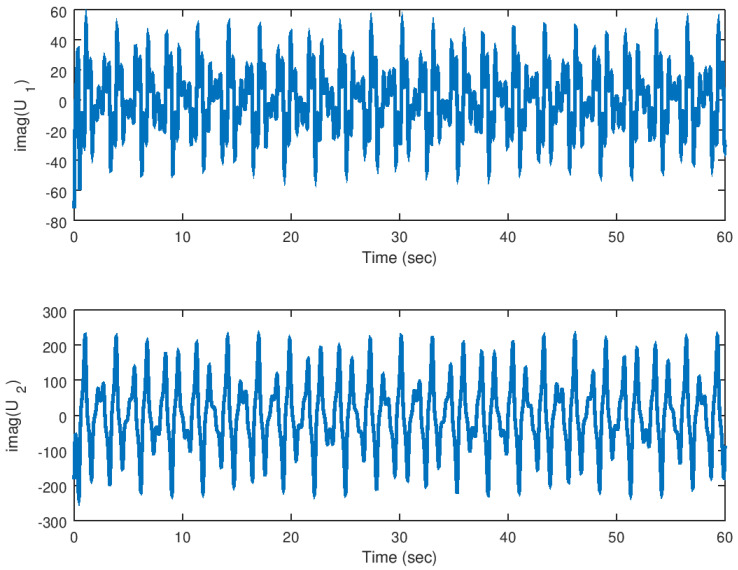
Evolution in time of the imaginary part of the input variables in the synchronization problem with complex parameter.

Finally, in Figure 18 and Figure 19, the input variables for this experiment are shown in which the real part and complex part are shown, respectively, evincing the control effort yielded by the proposed backstepping controller, and similar as the real valued parameter case, the necessary control input is provided to drive fast the error variables to zero.

## 6. Discussion

As observed in the theoretical and numerical results of this study, it is noticed that a new methodology to find hidden chaotic attractors is convincing even when the type of chaotic system is of complex variable. With this methodology, the hidden attractor is found directly in the complex domain by the help of the Bendixson theorem, which allows to find the parameter values in which one hidden chaotic attractor is found. It is important to clarify that other or multiple hidden chaotic attractors can be found in complex variable chaotic systems, and not only in this specific case in which a complex variable Lorenz chaotic attractor is analyzed. Besides, the stabilization backstepping technique provides control inputs that are tractable and simple something important when physical realization is needed. The backstepping technique provided in this research study, offers the virtual inputs to facilitate the recursive backstepping design avoiding the explosion of complexity phenomenon. The synchronization backstepping technique consider the error between the drive and response state variables, and even when the systems are identical in structure they are in different chaotic regimens, something important when these strategies would be implemented in real physical systems.

## 7. Conclusions

In this paper a stabilization and synchronization backstepping techniques for hidden chaotic attractors is presented. First, a hidden attractor is found in a complex variable Lorenz chaotic systems, something that offers a novel contribution to the field. The hidden attractor is found by implementing the set topology fundaments in order to find the intervals of the parameters of this chaotic system. Then, a backstepping controller and synchronizer are designed for their respective purposes. It is proved theoretically and numerically that the backstepping controller and synchronizer provide fast and accurate results. The importance of the design backstepping controller for the analyzed complex variable chaotic systems is that these results are valid for other kinds of chaotic and other types of nonlinear systems. As explained before, the importance and main contribution of this research study is that for the backstepping design complex variable Lyapunov functions are implemented, something that is not commonly found in the literature, so a complex control law is found appropriately. This results can be extended to backstepping control of hyper-chaotic systems, complex chaotic networks and distributed systems. In the future, these techniques will be extended to other kinds of complex chaotic systems.

## Data Availability

Not applicable.
